# Elixir of Vitriol and Tannin as a Haemostatic

**Published:** 1868-03

**Authors:** 


*Elixir of Vitriol and Tannin as a Haemostatic.*—The combination of elixir of vitriol and tannic acid, to which we called the attention of our readers in the February number of the Journal, has proved upon trial, a very convenient hæmostatic agent for dental use. It has been found very effective in internal as well as external hæmorrhages, and is more agreeable, when applied to the mouth, than many of the agents possessing like properties. Dr. A. P. Merill became aware of its value in 1857 in a case of tonsilitis, where owing to the morbid condition, a thin slice was pared from the surface of each tonsil. But little bleeding followed during the day but at night after the patient retired to bed a serious hæmorrhage occurred. Dr. M. on being summoned found the pulse feeble, breathing labored and strength greatly prostrated. The pulsations of the bleeding artery in one of the tonsils could be seen and there was an oozing of blood from the cut surfaces. Having no other remedy on hand Dr. M. made a mixture of elixir of vitriol and tannic acid, and applying it freely, the hæmorrhage was he states immediately and permanently arrested. It is also effective in diarrhœa where astringents are indicated. We recommend this preparation for hæmorrhages following the extraction of teeth, in two cases of which we have successfully used it.

